# Hyperspectral imaging for non-destructive prediction of fermentation index, polyphenol content and antioxidant activity in single cocoa beans

**DOI:** 10.1016/j.foodchem.2018.03.039

**Published:** 2018-08-30

**Authors:** Nicola Caporaso, Martin B. Whitworth, Mark S. Fowler, Ian D. Fisk

**Affiliations:** a Division of Food Sciences, University of Nottingham, Sutton Bonington Campus, LE12 5RD, UK; b Campden BRI, Chipping Campden, Gloucestershire GL55 6LD, UK; c 54 Towthorpe Road, Haxby, York YO32 3NA, UK

**Keywords:** AA, antioxidant activity, ABTS, 2,2′-azino-bis(3-ethylbenzothiazoline-6-sulphonic acid, CV, coefficient of variation (%), DPPH, 2,2-diphenyl-1-picrylhydrazyl, FI, fermentation index, HSI, hyperspectral imaging, NIR, near-infrared, PLS, partial least squares, RMSE, root mean square error, RMSEC, RMSE of calibration, RMSECV, RMSE of cross-validation, RMSEP, RMSE of prediction, RPD, ratio of performance deviation, TP, total polyphenols, TEAC, Trolox equivalent antioxidant capacity, *Theobroma cacao*, Hyperspectral chemical imaging, Cocoa quality, Antioxidant capacity, Phenolics, Near-infrared spectroscopy

## Abstract

The aim of the current work was to use hyperspectral imaging (HSI) in the spectral range 1000–2500 nm to quantitatively predict fermentation index (FI), total polyphenols (TP) and antioxidant activity (AA) of individual dry fermented cocoa beans scanned on a single seed basis, in a non-destructive manner. Seventeen cocoa bean batches were obtained and 10 cocoa beans were used from each batch. PLS regression models were built on 170 samples. The developed HSI predictive models were able to quantify three quality-related parameters with sufficient performance for screening purposes, with external validation R^2^ of 0.50 (RMSEP = 0.27, RPD = 1.40), 0.70 (RMSEP = 34.1 mg ferulic acid g^−1^, RPD = 1.77) and 0.74 (60.0 mmol Trolog kg^−1^, RPD = 1.91) for FI, TP and AA, respectively. The calibrations were subsequently applied at a single bean and pixel level, so that the distribution was visualised within and between single seeds (chemical images). HSI is thus suggested as a promising approach to estimate cocoa bean composition rapidly and non-destructively, thus offering a valid tool for food inspection and quality control.

## Highlights

• Measurements of single cocoa beans were made by NIR HSI.
• PLS regression models were built for several chemical properties.
• Fermentation (FI), total phenolics (TP) and antioxidant activity (AA) were predicted.
• Prediction performance was suitable for screening purposes.

## 1 Introduction

Cocoa phenolic compounds, antioxidant activity and fermentation index are important parameters for the understanding of cocoa bean quality. Non-destructive and rapid techniques for the analysis of granular food commodities are interesting for the food industry as well as for research laboratories. In addition, methods able to predict sample composition at an individual seed level may offer the advantage of understanding the natural variations in bean-to-bean composition.

Hyperspectral imaging (HSI) is a relatively novel technology in the field of food science and technology. It can analyse individual grain or bean samples in a rapid, non-destructive and non-contact manner and offers the possibility to scan samples at high throughput, also visualising the spatial distribution. Different configurations exist for data acquisition, with the “push-broom” approach being the most promising one for its practical applicability, as it acquires one spatial line at a time. The resulting image is commonly referred to as a “hypercube”, containing the two spatial dimensions and a third dimension represented by the spectra at each pixel. In food science, HSI has been mostly applied for the study of meat and fish products (ElMasry et al., 2012, Gowen et al., 2007), and less frequently used for granular food commodities (Moghaddam, Razavi, & Taghizadeh, 2013). HSI can be used for classification or quantification purposes, thus allowing a rapid identification of defective beans or seeds in a batch. An advantage over conventional Near-Infrared (NIR) spectroscopy is that HSI allows analysis at a single object level and not just bulk analysis. While NIR spectroscopy gives an average spectrum for the whole sample, HSI records a full spectrum for each pixel of the hypercube. Proper data processing and spectral pre-treatments are important in HSI, similarly to NIR spectroscopy. The pre-processing algorithms developed for NIR spectroscopy can be applied to HSI spectral data: for example, those developed to reduce unwanted light scattering effects (Burger and Gowen, 2011, Rinnan et al., 2009). Once a calibration has been developed by regression methods, HSI gives the possibility to apply it at a single pixel level, thus allowing the distribution of the predicted compound across a sample to be visualised. This approach has been recently applied to grains in the case of whole wheat kernels (Caporaso et al., 2017a, Caporaso et al., 2018a) and coffee beans (Caporaso et al., 2017b, Caporaso et al., 2018b).

Cocoa is the key ingredient for chocolate production. This paper investigated three quality-relevant constituents or properties of cocoa beans, namely polyphenols, antioxidant activity and fermentation index. Cocoa polyphenols are antioxidant compounds with beneficial health properties, and are responsible for the typical bitter taste of raw cocoa seeds (Oracz, Zyzelewicz, & Nebesny, 2015). Polyphenols are more abundant in fresh cocoa seeds than the fermented dried product, with an initial content up to 5–6% total phenolics (dmb). Further degradation of the polyphenol content can be observed when alkalisation is applied, together with changes in the colour of the nibs (cotyledons) (Fowler and Coutel, 2017). Niemenak, Rohsius, Elwers, Ndoumou, and Lieberei (2006) reported that the polyphenol content of cocoa beans varies from 67 to 149 mg g^−1^ (dmb) expressed as epicatechin equivalent in the freshly harvested product, with variation further depending on the botanical variety, geographical origin and postharvest processing.

Polyphenols have received attention because of their physiological effects including their antioxidant activity, i.e. the capacity to retard lipid oxidation. The antioxidant capacity of foods can be assessed by several methods, the most common being 2,2′-azino-bis(3-ethylbenzothiazoline-6-sulphonic acid) (ABTS) and 2,2-diphenyl-1-picrylhydrazyl (DPPH) assays. Solid foods have complex composition and contain both hydrophilic and lipophilic compounds, found in free or bound states, thus representing an issue for their extraction. In addition, polyphenols have different polarity, which further complicates the measurement of total antioxidant capacity. An approach recently proposed to overcome this issue, named QUENCHER (from “QUick, Easy, New, CHEap, and Reproducible”), involves the direct addition of free radicals to the solid food (Gökmen, Serpen, & Fogliano, 2009). ABTS or DPPH can be used to run the method, while ABTS was reported to be more sensitive to phenolic compounds and generally gives a slightly lower analytical error compared to DPPH (Serpen, Capuano, Fogliano, & Gökmen, 2007). The antioxidant activity of cocoa beans has been reported to vary depending on their origin (Othman, Ismail, Ghani, & Adenan, 2007). However, information on variation at a single cocoa bean level is not available.

Another important parameter related to cocoa bean quality is the fermentation level, which can affect the final flavour of cocoa powder and chocolate. Fermentation is often carried out by making a heap of fresh cocoa beans or placing them in wooden boxes for 3–7 days. The beans are then dried. An indication of the fermentation level can be obtained by visual assessment, using the cut test: unfermented beans are grey (slaty), under-fermented beans are violet or purple and fully fermented beans are brown in colour. However, this method is time consuming and subjective. Alternatively, a fermentation index (FI) can be obtained from an analytical determination that involves grinding the cocoa beans, pigment extraction using methanol:HCl and spectrophotometric readings at 520 and 460 nm. Despite the more objective measurement, this method is still time-consuming (overnight extraction) and involves the use of harmful reagents. Thus, rapid methods to predict fermentation index of cocoa, potentially on a single bean basis, would be of great practical interest.

Despite no work being published on within-batch cocoa seed variation, a large variability of chemical composition is expected, similar to that of other seeds or fruits. For other tree species, large natural variability was reported even within the same plant (Pannico et al., 2017). The post-harvest processing of cocoa beans (fermentation and drying) can further exacerbate natural differences, giving a wide range of properties within the same batch. For these reasons, the study of natural distribution of cocoa quality parameters such as phenolic compounds can be useful for the industry, to assess the homogeneity of a batch.

Relatively little research has been published for the quantification of cocoa constituents using non-destructive techniques. Examples include the application of traditional NIR spectroscopy for the prediction of fat content in ground cocoa (Álvarez et al., 2012, Teye and Huang, 2015, Veselá et al., 2007), as well as nitrogen compounds and moisture (Veselá et al., 2007), or minor compounds such as caffeine and theobromine (Álvarez, et al., 2012).

Other properties of cocoa beans have been estimated using FT-IR, in particular pH and fermentation index (Teye et al., 2015), with promising results. Phenolic compounds, and particularly procyanidins, have been investigated in cocoa mass (liquor) using NIRS (Whitacre et al., 2003). The spectral region used was 700–2500 nm and the reference measurement was carried out using HPLC analysis. Very good prediction was reported, with R^2^ value of 0.98, for total procyanidin oligomers.

Only one research paper used IR methods on whole cocoa beans for quantitative prediction. Sunoj, Igathinathane, and Visvanathan (2016) applied FT-IR (800–2778 nm) to build PLS regression models to predict total polyphenols (TP), pH and FI. The best PLS prediction model for polyphenol content had R^2^ = 0.84 and RMSECV = 0.93 mg g^−1^ using vector normalisation pre-treatment. Similarly, good prediction was obtained for fermentation index, with the best model showing R^2^ = 0.88 and RMSECV = 0.06.

Other papers reporting on NIR applied to cocoa beans used ground beans or cocoa mass, thus little investigation has been carried out on whole cocoa nibs. No information has been published using HSI or even traditional NIR spectroscopy for single cocoa beans. The hypothesis tested in the present research was that HSI is able to predict cocoa chemical constituents and properties on a single seed basis.

Therefore, the aim of the present work was to use hyperspectral imaging (HSI) in the spectral range 1000–2500 nm to predict fermentation index, total polyphenols and antioxidant activity of individual dry fermented cocoa beans. In addition, we report on the distribution of these compounds in a wide range of samples showing single-seed variability.

## 2 Materials and methods

### 2.1 Samples and reagents

All the reagents used were analytical grade. HPLC-grade methanol and ethanol, and HCl were supplied by VWR (Poole, UK). ABTS was supplied by Roche (Penzberg, Germany). Folin-Ciocalteu reagent, Ferulic acid (99% purity), (±)-6-Hydroxy-2,5,7,8-tetramethylchromane-2-carboxylic acid (Trolox; 97% purity) and potassium persulfate (99.99%) were supplied by Sigma-Aldrich (St. Louis, Missouri, USA).

Seventeen batches of fermented, unroasted cocoa beans were obtained from importers representing a range of producing countries: Brazil (1 batch), Cameroon (1), Ecuador (3), Ghana (4), Indonesia (1), Ivory Coast (3), Mexico (1), Nigeria (2) and Venezuela (1). Ten representative samples of sound unbroken cocoa beans were selected from each batch, thus obtaining 170 single beans, each bean representing a sample for the purposes of our work.

### 2.2 Hyperspectral imaging

The HSI system used was produced by Gilden Photonics Ltd. (Glasgow, UK), including a SWIR camera (Specim, Oulu, Finland) with a cooled 14 bit 320 × 256 pixel HgCdTe detector. 240 spectral bands were obtained covering the range 1000–2495 nm, with ∼6 nm resolution. Cocoa beans were analysed by HSI as shelled whole beans (cotyledons). Ten seeds at a time were placed on a movable plastic stage and scanned using the push-broom approach, at a frequency of 100 lines s^−1^. A lens of focal length 31 mm was used, giving a field of view of 35 mm, with a pixel size of 0.109 mm (320 pixels). Two 500 W incandescent lamps were used as the illumination source, oriented at 45°. A baseline correction was made for each image and a normalisation was made using a white (PTFE) reference to calculate absorbance hypercubes (Caporaso, Whitworth, & Fisk, 2017).

Image processing was done as reported by Caporaso et al., 2017a, Caporaso et al., 2018a, Caporaso et al., 2017b, Caporaso et al., 2018b using ENVI-IDL 5.2 (Exelis, USA), with in-house programs developed to perform specific operations such as absorbance calculation, bad pixel removal, object segmentation and average spectrum calculation. The average spectra for each cocoa seed were exported for chemometric analysis. Each seed was scanned on both sides, so that the final number of average spectra for the prediction models was 340. After scanning, the shelled cocoa beans were individually ground using a pestle and mortar, yielding approximately 1 g material for each bean. The samples were then stored at −20 °C, in readiness for reference analyses. A scheme of the experimental design is reported in Supplementary material 1.

### 2.3 Fermentation index

Fermentation index (FI) was measured according to the method of Sunoj et al. (2016). About 10 mg of ground, shelled cocoa beans were placed in glass tubes and 5 mL of a methanol:HCl (97:3 v/v) solution was added, and then stored at 10 °C for 20 h. The mixture was then filtered using a Whatman filter no. 1 and spectrophotometric readings were made at 460 and 530 nm using an Evolution 201 spectrophotometer (Thermo Fisher scientific, Waltham, WA, USA). The fermentation index is defined as the ratio between these absorbances (460/530), with values above 1 indicating a well fermented sample and values below 1 indicating under-fermented beans.

### 2.4 Total polyphenols

Total polyphenols (TP) were analysed according to the Folin-Ciocalteu method (Singleton & Rossi, 1965), as described by Othman et al. (2007), slightly modifying the volumes. The extraction conditions were chosen according to Sunoj et al. (2016) for cocoa bean phenolic analysis. In particular, the total polyphenols were analysed directly on the ground cocoa material.

1 mL of an ethanol:water solution (70:30 v/v) was added to a 100 mg sample of ground cocoa. The dispersion was shaken for 30 s and centrifuged for 5 min at 1500*g* using a Mikro 22 centrifuge (Hettich, Tuttlingen, Germany). 100 µL of the supernatant was taken and 750 µL of Folin-Ciocalteu reagent were added. After exactly 5 min, 750 µL of a 0.45 M sodium hydrogen carbonate solution was added to stop the reaction. After 90 min, the absorbance was read at 725 nm using an Evolution 201 spectrophotometer. A 7-point calibration curve was built using pure ferulic acid in the range 0–0.20 mg mL^−1^, with R^2^ = 0.993. The standard error (SE) was calculated as the ratio between the standard deviation of the measurements and the square root of the number of replicates. The SE for total polyphenol reference analysis was 3.23 mg Ferulic acid g^−1^ cocoa. A second indicator of the repeatability of the reference measurement is given by the coefficient of variation (CV), which was 3.68% for polyphenol content analysis.

### 2.5 Antioxidant activity using a modified QUENCHER approach

The antioxidant activity (AA) was evaluated using the QUENCHER approach, according to Serpen et al. (2007). This method is particularly useful to assess the antioxidant capacity of solid foods, particularly for those compounds that are bound in the food matrix and are insoluble, yet exert AA (Gökmen et al., 2009). The ABTS working solution was prepared by mixing a 0.7 mM aqueous solution ABTS and 2.45 mM potassium persulphate. The mixture was kept in the dark at room temperature for 12–16 h to obtain a stable ABTS solution. ABTS working solution was prepared by diluting (∼1:88) this ABTS standard solution with 50:50 ethanol:water (v/v). 10 mg of a ground cocoa bean were added to 1.7 mL ABTS working solution. After vortexing the samples for 2 min, they were placed in an orbital shaker and centrifuged at 9200*g* for 2 min. The supernatant was taken and absorbance at 734 nm was read using an Evolution 201 spectrophotometer. The reading was performed at exactly 6 min after mixing the ABTS reagent with the sample. Trolox was used to build a calibration curve to express the antioxidant capacity as Trolox equivalent antioxidant capacity (TEAC), i.e. mmol Trolox equivalent kg^−1^ cocoa. A 5-point calibration curve was built in the range 20–120 mM Trolox equivalent, showing R^2^ = 0.978. The SE for this method was 21.02 mmol Trolox equivalent kg^−1^ cocoa, with CV value of 14.78%.

### 2.6 Data treatment and statistical analysis

Of the 340 average spectra obtained from single cocoa beans, 240 were used for the calibration set, while 100 were used for the validation set. From each batch (10 beans), 70% of the beans were randomly selected to be part of the calibration set, while the remaining 30% were used as the validation set. The two average spectra belonging to a single cocoa bean were both included either in the calibration or in the validation dataset. The spectral data exported for each cocoa bean were treated in The Unscrambler 10.3 (CAMO, Norway). Several spectral pre-treatment algorithms were tested to remove light scattering interferences and other noise, including area normalisation, Standard Normal Variate (SNV), and first and second derivative treatment using Savitzky-Golay smoothing (5 smoothing points, second order polynomial). Partial Least Squares (PLS) regression was applied to build quantitative models, using the spectral range 1000–2500 nm, corresponding to 240 bands. The optimal number of latent variables (LVs) in the PLS was chosen by leaving the software to suggest the best number of latent variables (based on the amount of explained variance and the prediction error in relation to the number of latent variables), which is commonly carried out to reduce the cross-validation prediction error, while avoiding model overfitting. In particular, models using a full cross-validation on the calibration datasets were built, the prediction error was evaluated as a function of the number of LVs, and the model with the minimum root mean square error of calibration (RMSECV) was selected. Once the optimal number of LVs had been established, further validation was carried outbuilding new models using the external validation dataset. The performance of the prediction models was tested by evaluating the coefficient of correlation (R^2^), the root mean square error of calibration (RMSEC) and prediction (RMSEP), as well as the Ratio of Performance to Deviation (RPD) (Fearn, 2002). In addition, Pearson correlation was used to verify the presence and strength of correlations among the parameters analysed, by setting values of significance at P < 0.05 (significant) and P < 0.01 (highly significant).

## 3 Results and discussion

### 3.1 Analysis of polyphenols, antioxidant activity and fermentation index

**Fig. 1:**
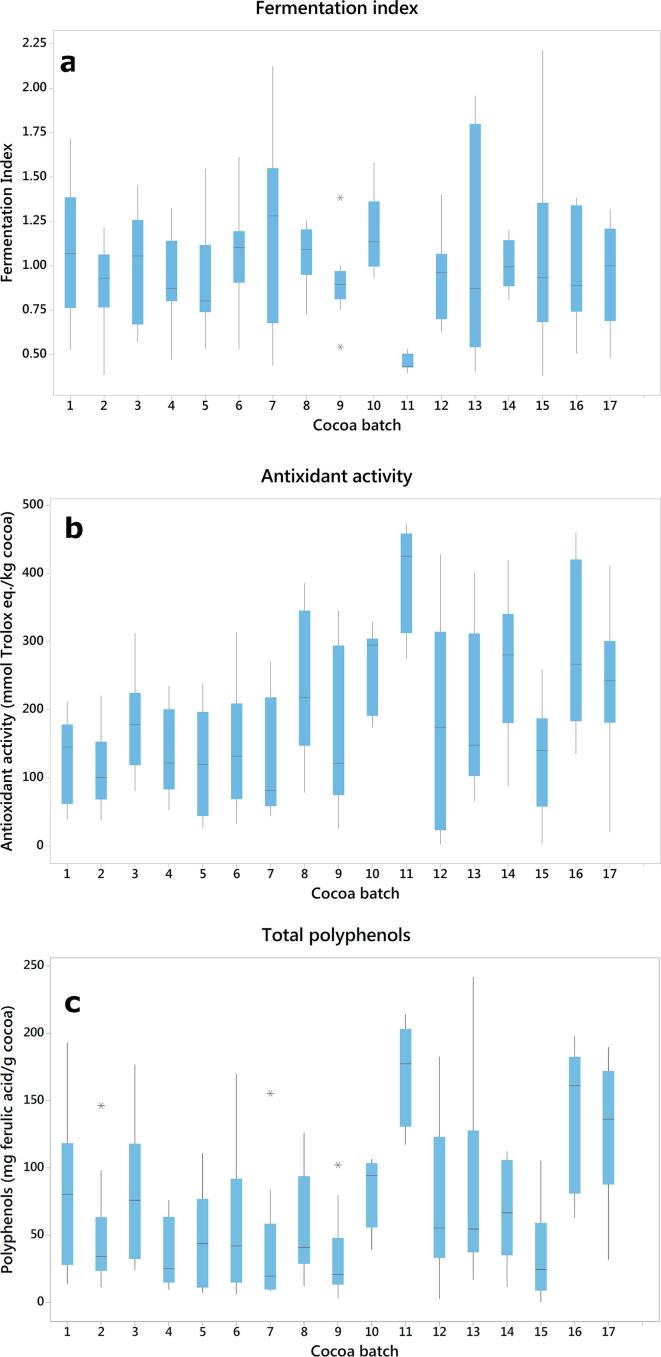
Distribution of (a) fermentation index, (b) antioxidant activity and (c) polyphenol content in dry fermented cocoa beans assessed on a single cotyledon basis (n = 10 per batch). Boxes represent the interquartile range, vertical bars indicate the maximum and minimum values, horizontal bars represent the median, while asterisks indicate potential outliers.

Reference measurements for total phenolic compounds, antioxidant activity and fermentation index for single, shelled cocoa beans are shown in Table 1. For the calibration set, fermentation index ranged from 0.38 to 2.13, with an average FI of 0.98 and standard deviation of 0.34. Antioxidant activity ranged from 13.8 to 462.7 mmol Trolox equivalent kg^−1^ cocoa, mean 186.1 and standard deviation (SD) of 116.1 mmol Trolox equivalent kg^−1^ sample. Average polyphenol content was 68.4 mg ferulic acid equivalent g^−1^ cocoa, with a standard deviation of 54.5, a minimum 2.9 and maximum 204.5 mg ferulic acid equivalent g^−1^ cocoa. The reference methods used in the present study are well established procedures, and the parameters analysed are accepted by the industry as cocoa quality indicators. The direct measurement of total polyphenols on the cocoa beans was previously reported by Sunoj et al. (2016) and was suggested as a valid approach. In our case, we analysed cocoa bean composition on a single seed basis, thus giving further information on the possible natural variability within a batch. Fig. 1 shows the intra- and inter-batch variability of the three parameters studied, where each batch consists of 10 single cocoa beans. A wide variability within and between batches was obtained for the three parameters evaluated, and in several cases one single batch almost covered the full range for the total samples. The within-batch distribution strongly depended upon the batch, for example in three batches (7, 13 and 15) the fermentation index was very broad, suggesting little uniformity during fermentation, or mixtures of well-fermented beans with poorly fermented ones. On the other hand, one batch (11) had a very low range of fermentation indices, as this batch consisted of under fermented Ecuadorian beans. This batch also showed the highest values of AA and TP. These results are relevant for practical applications, as obtaining a consistent and uniform quality of the raw material is important for the production of high quality end-products.

**Table 1:** Descriptive statistics for the parameters evaluated in single cocoa beans. Fermentation index is expressed as absolute values, while polyphenols are expressed as mg ferulic acid equivalent g^−1^ cocoa; antioxidant activity is expressed as mmol Trolox equivalent kg^−1^ cocoa.

Parameter	Calibration (n = 238)				Validation (n = 102)			
	Min	Max	Mean	SD	Min	Max	Mean	SD
Fermentation index	0.38	2.13	0.98	0.34	0.39	2.21	1.00	0.38
Total polyphenols	2.9	204.5	68.4	54.5	6.4	213.9	85.5	60.5
Antioxidant activity	13.8	462.7	186.1	116.1	20.9	472.3	208.8	114.9

SD = standard deviation.

Correlations between the parameters analysed were tested. TP and AA had a strong statistical correlation (P < 0.001), with Pearson r = 0.876. Similarly, a strong statistical correlation was observed between polyphenols and fermentation index, with r = −0.570, and between FI and AA, r = −0.443. These results were expected, as polyphenols are known to be a major source of the antioxidant activity in many food products. In addition, a strong decrease of polyphenols is expected to occur during cocoa bean fermentation, from an initial content of 150–200 mg g^−1^ cocoa (fat free) in fresh beans (Fowler & Coutel, 2017), to 60–100 mg g^−1^ expressed as ferulic acid equivalent, in fully fermented seeds (Jonfia-Essien, West, Alderson, & Tucker, 2008).

FI is a parameter commonly assessed in research laboratories and at the industrial level, as the degree of fermentation is related to differences in composition. For example, fermentation causes dramatic changes in the phenolic profile. Estimation of the degree of fermentation is commonly carried out by assessing the colour changes inside the cocoa bean, or the use of phenolic compounds as an indication of fermentation, as well as using fermentation index as an analytical parameter (León-Roque et al., 2016, Romero-Cortes et al., 2013). Anthocyanins exhibit a maximum absorbance at 500–550 nm and their concentration decreases during fermentation. The ratio 460/530 nm of a cocoa methanolic acid extract has been shown to be a reliable indicator of fermentation index, demonstrating a non-linear relationship with the sensory cut-test (León-Roque et al., 2016). The FI of cocoa beans has been previously correlated with total polyphenol content, which starts to decrease significantly over fermentation as soon as 72 h time. Fermentation index has been shown to increase continuously over fermentation time (Romero-Cortes et al., 2013).

Polyphenol content in cocoa is an important quality parameter, as it affects the bitterness and astringency of the final product, as well as its health properties. Despite the inconsistency often found in the literature about the polyphenol content of cocoa beans, a recent review reported the data published by several sources, and described the content as ranging from 40 to about 150 mg g^−1^ cocoa (Oracz et al., 2015). Previous works demonstrated that the level of TP decreases as function of fermentation time, e.g. Bonvehi and Coll (1997) reported a decrease from approximately 150 to less than 50 mg g^−1^ cocoa.

Our mean results are in agreement with these findings, in terms of average TP. The cocoa with the minimum phenolic content (9) had 33.1 mg polyphenol g^−1^ cocoa, while the highest value was observed for batch 11 which related to under fermented beans, and contained 166.7 mg TP g^−1^ cocoa. When looking at single bean variability, a large variation was observed, reaching very low values in several cases, while the highest value was approximately 200 mg g^−1^ cocoa.

Ortega et al. (2008) investigated different fractions of phenolic compounds and the level of TP in whole cocoa beans (including the shell), nibs (i.e. broken cotyledons), cocoa mass and powder, reporting concentrations of ∼140 mg g^−1^ in the cocoa powder, and ∼250–300 mg g^−1^ in the other sources. The highest value was observed for cocoa nibs, with higher concentrations than for beans due to the dilution effect of the shell. Lower levels were found in the cocoa mass and powder probably due to degradation during processing. Previous literature also suggested the absence of qualitative difference among different genotypes of cocoa or fermentation processes, but dramatic quantitative differences are often observed, as a result of growing and fermentation conditions (Niemenak et al., 2006). The authors investigated individual phenolic compounds and total polyphenols in several cocoa clones under two fermentation-like conditions. The total polyphenols ranged from ∼80 to 140 mg g^−1^, with some compounds showing interesting larger variability, e.g. 2.4–44.6 mg g^−1^ for catechin and 0.15–2.8 mg g^−1^ for cyanidin-3-galactoside.

Unfermented dried beans have very high phenolic content, up to 20%, which is mainly represented by proanthocyanidins, flavan-3-ols and anthocyanins (Afoakwa, 2016). However, during fermentation, oxidation and polymerisation (condensation) reactions take place with consequent significant decrease in phenolic content, and there is some diffusion of phenolic compounds into the fermentation sweatings (Wollgast and Anklam, 2000).

### 3.2 HSI for prediction of whole cocoa bean composition

**Fig. 2:**
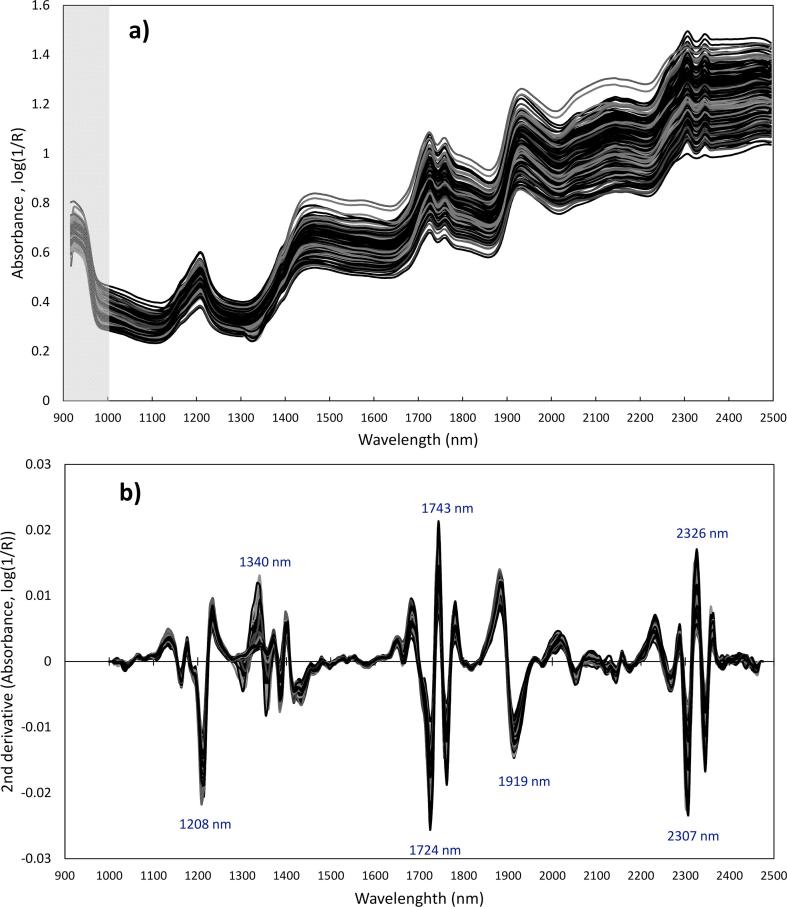
Average spectra of single shelled cocoa beans (170 cotyledons, 340 spectra), (a) showing the absorbance without spectral pre-treatments and (b) treated by applying second derivative calculation. The region in grey (below ∼990 nm) does not contain useful spectral data for this camera detector, and was excluded.

The spectra of cocoa beans acquired by HSI for individual cotyledons are reported in Fig. 2. Spectral pre-processing was applied to remove non-chemical effects such as baseline and slope variation. Fig. 2(b) shows the 2nd derivative treated spectra. Significant absorbance bands are seen at 1208, 1340, 1397–1437, 1724–1743, 1919 and 2307–2326 nm. The absorption band at 1208 nm is probably due to the C–H stretching second overtone (–CH_3_ or –CH_2_), attributed to carbohydrates (Esteban-Dıez et al., 2004, Osborne et al., 1993. Absorbance at 1397 nm has been previously assigned to second C–H stretching and C–H deformation, corresponding to the –CH_2_ structure. Similarly, around 1440 nm, the C–H combination is exhibited. The wavelength 1919 nm has been attributed to the C=O stretching second overtone in the carbonyl groups (–CO_2_H or CONH) (Esteban-Dıez et al., 2004, Osborne et al., 1993). However, this absorption band is very close to 1923 nm, which is assigned to the O–H group of water (Workman & Weyer, 2012), and therefore it might be influenced by this group. The HSI absorbances at 1724–1743 and 2307–2326 nm are characteristics of lipids, and the clear peaks observed at these wavelengths are justified by the high fat content of the cocoa beans. The absorption at 1744 nm has been previously assigned to lipids, as C–H stretch first overtone (CH_2_) absorbs at this wavelength. The CH_2_ group also absorbs at 1725 nm, due to the C–H stretch first overtone (Burns and Ciurczak, 2007, Osborne et al., 1993).

PLS regression was used to build prediction models. The performance of the predictive models is shown in Table 2, depending on the spectral pre-processing applied.

**Table 2:** Performance of the PLS regression models for fermentation index, total polyphenols and antioxidant activity in single cocoa beans obtained by HSI in the spectral region 1000–2500 nm.

	LV	Calibration		Cross-validation			Prediction			Slope	Bias
		R_c_^2^	RMSEC	R_cv_^2^	RMSECV	RPD_cv_	R_p_^2^	RMSEP	RPD_p_		
*Fermentation index*											
Log(1/R)	15	0.534	0.230	0.502	0.249	1.37	0.489	0.275	1.38	0.457	−0.047
Normalisation	16	0.570	0.223	0.523	0.243	1.40	0.498	0.272	1.40	0.474	−0.037
SNV	14	0.536	0.232	0.516	0.234	1.45	0.454	0.283	1.34	0.449	−0.062
1st derivative	11	0.492	0.242	0.475	0.255	1.33	0.355	0.308	1.23	0.362	−0.016
2nd derivative	8	0.470	0.243	0.360	0.282	1.21	0.300	0.321	1.18	0.310	−0.017

*Total polyphenols*											
Log(1/R)	15	0.809	23.96	0.727	29.74	1.83	0.660	36.39	1.66	0.677	−6.12
Normalisation	16	0.817	23.35	0.757	28.09	1.94	0.700	34.13	1.77	0.665	−7.06
SNV	14	0.812	23.66	0.660	36.49	1.49	0.680	35.21	1.72	0.652	−3.30
1st derivative	11	0.820	22.98	0.744	28.81	1.89	0.551	41.84	1.45	0.652	−13.90
2nd derivative	8	0.810	23.39	0.697	31.36	1.74	0.573	41.04	1.47	0.591	−13.31

*Antioxidant activity*											
Log(1/R)	15	0.818	49.37	0.710	62.72	1.85	0.625	71.15	1.62	0.810	−9.79
Normalisation	16	0.825	48.25	0.738	59.55	1.95	0.680	65.88	1.74	0.804	−13.33
SNV	14	0.804	51.11	0.664	67.37	1.72	0.652	68.44	1.68	0.766	−4.35
1st derivative	11	0.831	46.97	0.747	58.52	1.98	0.646	69.10	1.66	0.899	−24.45
2nd derivative	8	0.764	55.25	0.741	59.23	1.96	0.735	60.04	1.91	0.758	−11.96

LV = latent variables. R^2^ = coefficient of regression. RMSEC, RMSECV and RMSEP = root mean square error of calibration, cross-validation and prediction, respectively. RPD = ratio of performance deviation. Total polyphenols is expressed as mg ferulic acid equivalent g^−1^ cocoa; antioxidant activity is expressed as mmol Trolox equivalent kg^−1^ cocoa. The same number of LV was applied for the calibration, cross-validation and prediction.

Good prediction of cocoa bean composition was achieved. However, the model for FI was less strong, although it could be acceptable for separating high and low FI samples (prediction R^2^ = 0.498 and RMSEP = 0.272, with RPD of 1.40). Better performance was achieved for TP and AA. Area normalisation pre-processing gave the best prediction for TP, with R_c_^2^ = 0.817 and R_p_^2^ = 0.700; the prediction error was RMSEC = 23.4 and RMSEP = 34.1 mg Ferulic acid equivalent g^−1^. The best performance was achieved for AA prediction, using second derivative treatment, which gave R_c_^2^ = 0.764 and R_p_^2^ = 0.735. The calibration error was 55.3 and the prediction error was 60.0 mmol Trolox kg^−1^ cocoa.

The performance of TP and AA is sufficient for practical use, as the RPD values of 1.8 and 1.9 are almost comparable to previous work using traditional bulk NIR spectroscopy techniques for whole cocoa beans. However, as expected for whole beans the models are poorer than those previously published for ground cocoa or cocoa mass, due to the less homogenous sample presentation, less uniform illumination, curved sample surface and poorer detector characteristics. However this is compensated for by the additional flexibility and applicability of HSI.

**Fig. 3:**
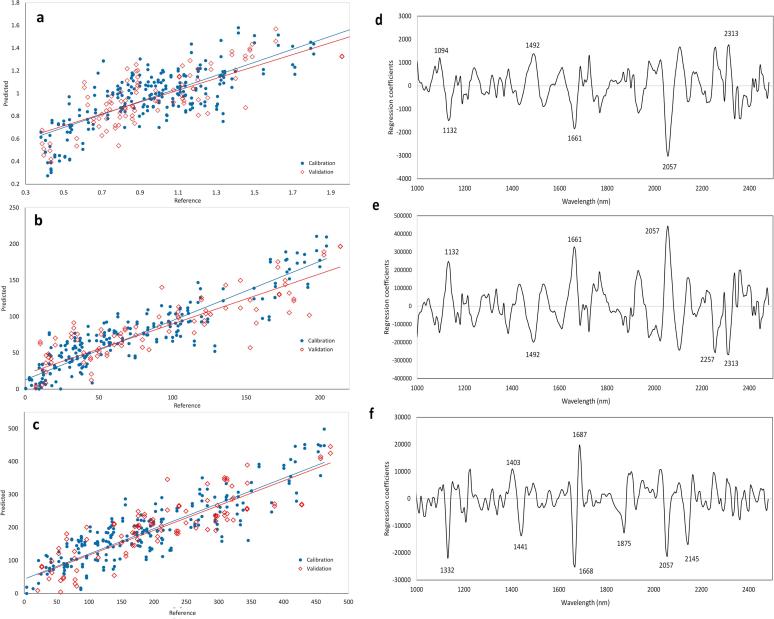
Left: Predicted vs reference (a) fermentation index, (b) total polyphenols, and (c) antioxidant activity in single shelled cocoa beans, by applying the best PLS calibration using HSI. Right: Regression coefficients of the best PLS models for quantitative prediction of (d) fermentation index, (e) total polyphenols and (f) total antioxidant activity. The numbers indicate the wavelengths expressed in nm.

Fig. 3 shows the predicted vs reference FI, TP and AA, for the best calibration models, by separately showing the calibration and the external validation datasets, and the PLS regression coefficients for these predictions, with indication of the most important wavelengths. Some wavelengths had strong influence on several models, for example those at 1132, 1492, 1661, 2057 and 2313 nm. In addition, FI was also influenced by the wavelength at 1094 nm and AA was also influenced by the wavelengths at 1403, 1441, 1687, 1875 and 2145 nm. Absorbance around 1490 nm has been attributed in the literature to several possible chemical bond vibrations, including N–H stretch first overtone and O–H stretch first overtone, thus indicating amides or compounds such as cellulose. The absorbance around 1400 nm is caused by the C–H combination bond, thus indicating a –CH_2_ structure. However, absorbances around 1450 nm have been attributed to carbonyl groups (C=O, e.g. ketones and aldehydes) as well as O–H polymeric groups, which can be due to complex carbohydrates (Burns & Ciurczak, 2007). Interestingly, the region 1400–1440 nm is important for the three models, and this region has also been attributed to aliphatic alcohols and to phenols, even monomeric phenols (which absorb at 1420 nm) (Workman & Weyer, 2012). The absorbance at 2057 nm observed for all the models indicates an N–H stretch/amide 1st combination band, which has been attributed to protein. The peaks at 2145 and 2313 nm have been tentatively attributed to C–H deformation and C–H deformation and C–H bend second overtones respectively, both indicating lipids (Burns & Ciurczak, 2007). The influence of lipid absorbances in the models for TP and AA might be explained by their possible negative influence on these parameters due to the fact that beans with higher relative fat content have lower non-fat solids, where polyphenols are concentrated.

Limited information has been found in the literature regarding the prediction of polyphenol content using NIR-based techniques, and HSI has not been tested for cocoa bean analysis.

For other agricultural commodities, similar large heterogeneity in polyphenol content was reported by Nogales-Bueno, Baca-Bocanegra, Rodríguez-Pulido, Heredia, and Hernández-Hierro (2015), who reported considerable variation in single grape berries. The authors applied HSI in the spectral range 900–1700 nm to scan two grape varieties, collecting 100 single berries per variety which were used for three different reference analyses, so the final calibrations consisted of 33 samples. The PLS prediction model built using the Folin-Ciocalteu quantification had R^2^ = 0.82 and SECV = 0.92 mg g^−1^ (range: 1.4–4.2 mg g^−1^).

The results herein presented are comparable with previous reports where NIRS was used for the prediction of polyphenol content and antioxidant activity. For example, Catelani et al. (2017) recently reported NIRS calibrations for roasted coffee beans, with validation R^2^ = 0.927 and 0.909 for polyphenol content and antioxidant activity. The RMSEP was 1.97 (range: 25–50) g gallic acid/kg coffee, and 95 (400–1250) mmol Trolox kg^−1^ of coffee, respectively.

The possibility of predicting fermentation index in cocoa beans using colour measurement has been recently reported by León-Roque et al. (2016). Two cocoa varieties were fermented in three different regions of Peru and sun-dried, before 40 beans per batch were sampled and manually shelled. The colour analysis was carried out using a mobile phone, “under constant conditions of light”. It has been reported that colour measurement by itself is not a useful way to predict fermentation index, and even the application of complex statistical models such as Artificial Neural Network (ANN) did not lead to very good prediction of FI based on colour.

Other authors have proposed different indicators of fermentation degree, in particular the amount of nitrogen compounds, and some authors also proposed a traditional NIR spectroscopy approach to estimate the concentration of NH_3_. Very good performance was shown for this parameter, with R^2^ = 0.97, SECV = 24 ppm (range 25–441) and RPD = 3.9, however the NIR spectra were acquired from bulk ground samples of cocoa beans (Hue et al., 2014). Therefore, no indication has been reported so far on single bean variability, especially predictions that avoid the grinding step of the seeds.

### 3.3 Visualisation of chemical images

**Fig. 4:**
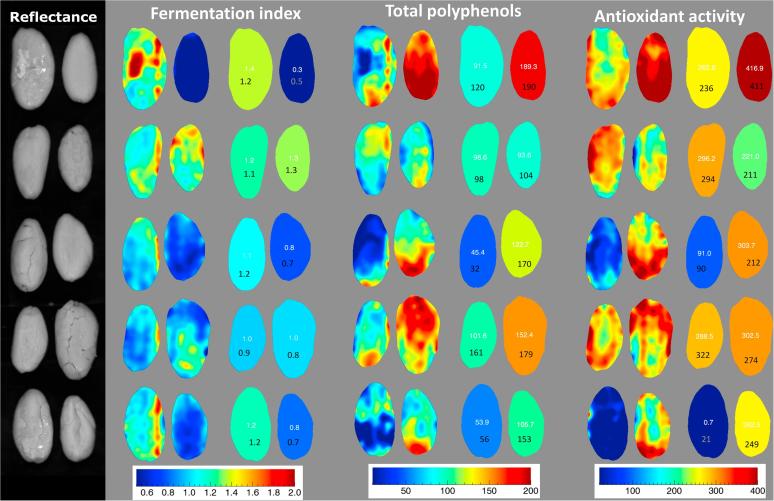
Applied calibrations (chemical images) for fermentation index, total polyphenol content and antioxidant activity in shelled cocoa beans. Calibrations are shown at a single pixel level (pictures on the left), or averaging the predicted values to obtain single-bean values (beans on the right). Numbers in white indicate the predicted average value for each cocoa bean, while those in black indicate the reference measurement. Fermentation index values are dimensionless, total polyphenols are expressed as mg ferulic acid equivalent g^−1^ cocoa, while antioxidant activity is expressed as mmol Trolox equivalent kg^−1^ cocoa.

Once calibrations had been developed for cocoa constituents, they were applied by HSI. It is possible to apply the prediction models for single cocoa beans to estimate the total phenolic content, antioxidant activity and fermentation index in a rapid way. In addition, HSI allows visualisation of the composition of a target compound or property within single beans, by applying the calibration at a single pixel level, as shown by an example in Fig. 4. Good prediction was obtained on shelled seeds, where the best HSI calibrations allowed accurate visualisation of the three parameters studied. Seeds with very low FI also showed the highest TP content and AA. Thus, by applying the proposed method, identification of cocoa seed quality can be made at single seed level, and visualisation of surface properties and the distribution of chemical composition may aid prediction of poor quality or damage. It should be also noted that prediction model for FI was quite poor, thereofore the chemical image for this attribute is for demonstration purpose only.

## 4 Conclusions

We report, for the first time, quantitative predictions based on HSI for whole shelled cocoa beans on a single bean basis. We used a wide range of samples to develop a comprehensive model which includes much of the expected variation in commercial cocoa beans. Our research demonstrated for the first time that estimation or prediction of fermentation index, total polyphenols and antioxidant activity is possible by NIR-HSI on a single cocoa bean level, thus allowing cocoa beans to be individually analysed without any previous grinding or extraction process. In addition, the method can be applied at a single pixel level, with potential to provide information on the distribution of composition within beans.

The bean-to-bean variability was found to be particularly wide, possibly due to the natural variation within and among plants due to environmental conditions, but also to the post-harvest practice. Our approach can be of interest for the scientific community, as it can be used to further elucidate the consistency of the fermentation/drying process to obtain higher quality product. In addition, it can also be used to identify cocoa nibs with particularly high or low polyphenol content or antioxidant activity in a non-destructive manner, which could in turn lead to the production of functional foods with increased nutritional properties, and improved product consistency.

## Appendix A Supplementary data

Supplementary data 1 Flow chart of the experimental design used for the non-destructive prediction of cocoa bean quality.
