# Supplementary figures and images for: Hyperspectral imaging for non-destructive prediction of fermentation index, polyphenol content and antioxidant activity in single cocoa beans
Source: Food Chem. 2018 Aug 30;258:343–51. doi: 10.1016/j.foodchem.2018.03.039 (PMC5914545; doi:10.1016/j.foodchem.2018.03.039)

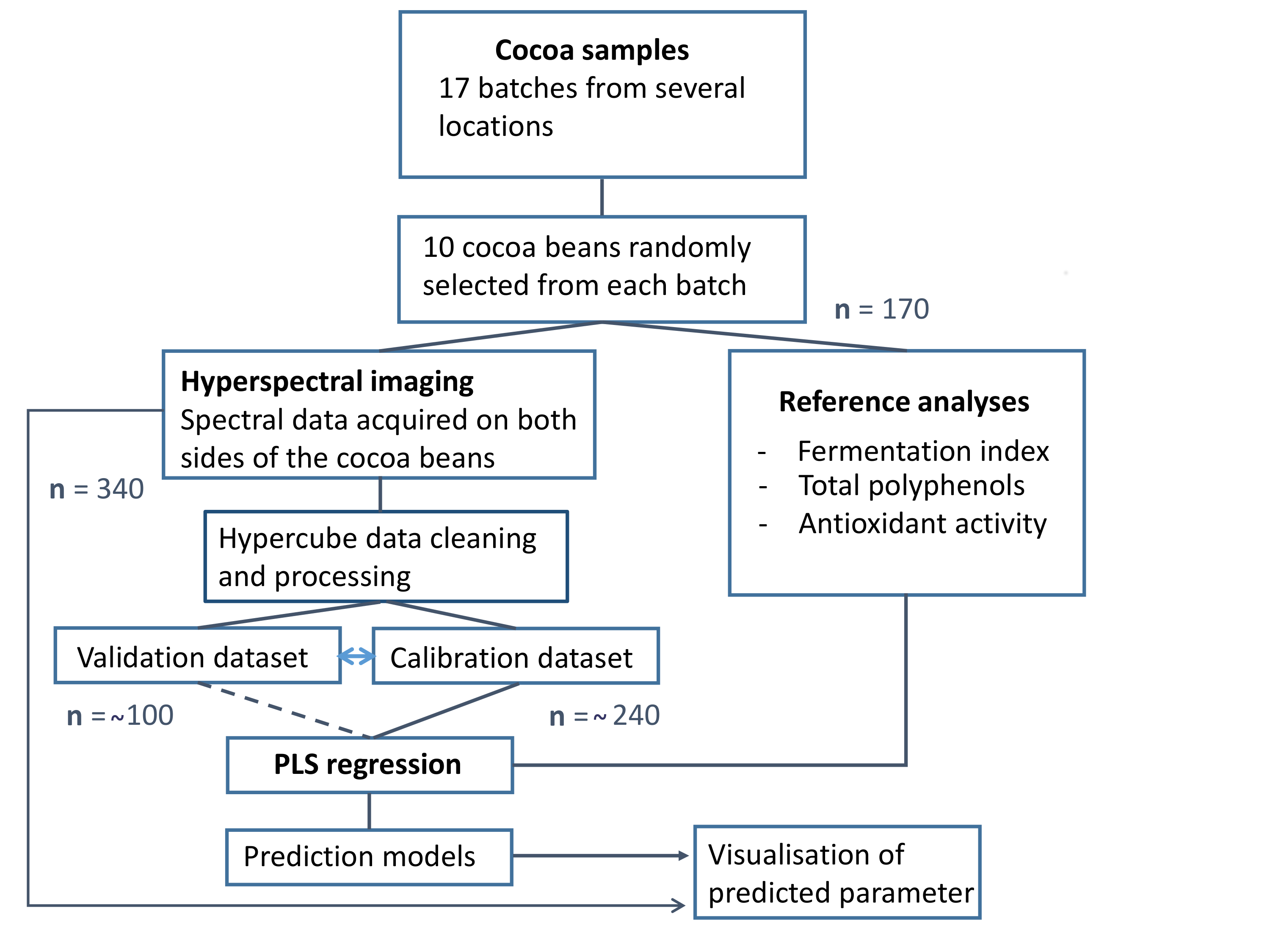

Supplement: Supplementary data 1 — Flow chart of the experimental design used for the non-destructive prediction of cocoa bean quality. [file mmc1.zip › Additional figure OK.png]
